# Differences and similarities in personality functioning across different types of eating disorders

**DOI:** 10.3389/fpsyt.2023.1155725

**Published:** 2023-06-01

**Authors:** Jens Rohde, Alexander Obbarius, Barbara Voigt, Lea Sarrar, Silke Biesenthal-Matthes, Clara-Sophia Kind, Matthias Rose, Tobias Hofmann

**Affiliations:** ^1^Department of Psychosomatic Medicine, Center for Internal Medicine and Dermatology, Charité – Universitätsmedizin Berlin, Corporate Member of Freie Universität Berlin and Humboldt-Universität zu Berlin, Berlin, Germany; ^2^Dornsife Center for Self-Report Science, University of Southern California, Los Angeles, CA, United States; ^3^Department of Psychology, Faculty of Sciences, Medical School Berlin, Berlin, Germany; ^4^Department of Psychosomatic Medicine and Psychotherapy, Gemeinschaftskrankenhaus Havelhöhe, Berlin, Germany; ^5^Department of Psychosomatic Medicine and Psychotherapy, Kliniken im Theodor-Wenzel-Werk, Berlin, Germany; ^6^Quantitative Health Sciences, Outcomes Measurement Science, University of Massachusetts Medical School, Worcester, MA, United States; ^7^Department of Psychosomatic Medicine and Psychotherapy, DRK Kliniken Berlin Wiegmann Klinik, Berlin, Germany

**Keywords:** eating disorder (ED), anorexia nervosa, purging type, restricting type, bulimia nervosa, operationalized psychodynamic diagnosis (OPD), personality functioning

## Abstract

**Objective:**

The classification of anorexia nervosa (AN) into subtypes is relevant due to their different symptomatology. However, subtypes (restricting type: AN-R; purging type: AN-P) differ also in terms of their personality functioning. Knowledge about these differences would allow for better treatment stratification. A pilot study indicated differences in structural abilities that can be assessed by the operationalized psychodynamic diagnosis (OPD) system. The aim of this study was therefore to systematically explore differences in personality functioning and personality between the two AN subtypes and bulimia nervosa (BN) using three personality (functioning) constructs.

**Methods:**

A total of *N* = 110 inpatients with AN-R (*n* = 28), AN-P (*n* = 40), or BN (*n* = 42) were recruited in three clinics for psychosomatic medicine. Assignment to the three groups was performed using a comprehensive questionnaire validated for diagnostic purposes (Munich-ED-Quest). Personality functioning was examined using OPD Structure Questionnaire (OPD-SQ), personality by using the Personality Inventory for DSM-5–Brief Form and Big Five Inventory-10. (M)ANOVAs were used to examine differences across eating disorder groups. In addition, correlation and regression analyses were conducted.

**Results:**

We observed differences on several sub- and main scales of the OPD-SQ. Whereas patients with BN showed the lowest levels, AN-R patients displayed the highest levels of personality functioning. On some sub- and main scales, such as “affect tolerance,” the subtypes of AN differed from BN, whereas on the scale “affect differentiation,” AN-R, differed from the other two groups. The total eating disorder pathology score of the Munich-ED-Quest best predicted overall personality structure [stand. β = 0.650; *t*(104) = 6.666; *p* < 0.001] and self-regulation [stand. β = 0.449; *t*(104) = 3.628; *p* < 0.001].

**Discussion:**

Our findings confirm most of the results of the pilot study. These findings can facilitate the development of stratified treatment approaches for eating disorders.

## 1. Introduction

Anorexia nervosa (AN) is a serious health condition that affects various areas of a persons’ life. It remains the mental disorder with the highest mortality rate (1). About one third of AN patients are severely ill, which means that they stay chronically ill and suffer from extensive short- and long-term complications (2–4). Although less lethal, Bulimia nervosa (BN) can also have considerable impact on a person’s physical, mental and social condition. Among the mental factors that contribute to the emergence and persistence of eating disorders are personality factors, similar to other mental disorders. For example, comorbid personality problems such as Borderline personality disorder are frequently reported in eating disorders (5). The relevance of these factors for diagnosis and treatment of eating disorders, however, remains a subject of ongoing debate. For example, in 2006, the Cognitive-interpersonal maintenance model of anorexia nervosa was introduced and updated with more empirical evidence in 2013 (6). This model assumes that cognitive, socio-emotional, and interpersonal elements interact with each other to both cause and maintain eating disorders. A feature of this model is that there are predisposing (personality) traits such as obsessive compulsive traits and anxious avoidance that increase the vulnerability to AN.

Despite continuous development of new and effective treatment approaches for eating disorders (ED) including Dialectical Behavior Therapy [DBT-E; (7)], Mentalization-based Treatment [MBT-ED; (8)], Maudsley Model of Anorexia Nervosa Treatment for Adults [MANTRA; (9)], and Focal Psychodynamic Psychotherapy [FPT; (10)], treatment outcome remains unsatisfactory (11, 12). The question therefore remains as to what the factors are that prevent successful treatment in eating disorders. Reasons for treatment failure are diverse and high dropout rates are observed in almost all treatment modalities (13). In part, dropout might be explained by impairments in interpersonal communication skills (14, 15) which is a common symptom of personality disorders (PD), especially in those associated with ED (5, 16, 17).

Beyond attachment styles and disordered mentalization, which both are PD associated constructs (18), personality has been broadly targeted in eating disorder research. Farstad et al. (17) found that patients with restrictive AN (AN-R) were most likely to exhibit an obsessive-compulsive, avoidant or dependent PD whereas obsessive-compulsive, avoidant, dependent, borderline, and paranoid PDs were most prevalent in purging type AN (AN-P) and in BN. However, even if PDs are common in patients with EDs, certainly not every patient with an ED has a comorbid PD (5) in terms of a categorizable mental disorder. PDs may be just the “tip of the iceberg” of impaired personality functioning in patients with EDs. A closer look at “subsyndromal” maladaptive personality functions might be informative for further meaningful stratification of ED patients.

In recent years, construct and classification of PDs have been extensively discussed (19). It has become clear that the majority of researchers and clinicians favor a dimensional classification of PDs over the traditional categorical classification. This paradigm shift in the conceptualization of personality disorders has already been incorporated into the Diagnostic and Statistical Manual of Mental Disorders (DSM-5) in the form of the Alternative Model of Personality Disorders (AMPD) and into the 11th revision of the International Classification of Diseases (ICD-11).

**FIGURE 1:**
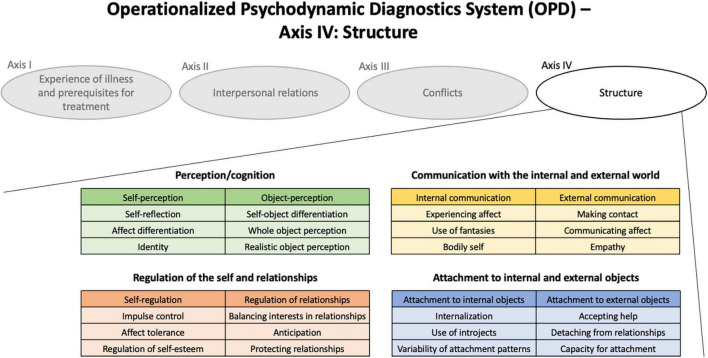
The OPD-System and its fourth Axis: Structure.

Another dimensional approach that has a broad overlap with the DSM-5 AMDP and ICD-11 PD classifications is the structural axis of the Operationalized Psychodynamic Diagnosis [OPD, (20)] system. It is the fourth axis of the OPD system. Personality structure, as operationalized in the OPD, is a construct very similar to the levels of personality functioning (LPFS) as introduced in DSM-5 (21). The OPD personality structure axis is a scale based on four basic functions (perception/cognition, regulation, communication, and attachment) differentiated in functions that relate to either the self, or the others. Thus, it describes eight dimensions which are categorized into three subdimensions each (24 subdimensions in total) that capture basic and clinically meaningful psychological abilities usually acquired in childhood and adolescence (see Figure 1 for an overview).

The system of the OPD personality structure therefore allows not only to quantify overall personality functioning dimensionally, but also to describe the areas of personality functioning in more detail. It has proven its value as a valuable tool to capture impairments of personality function that do not reach the threshold of a manifest personality disorder and to make them useful for diagnostic and therapeutic purposes (22).

Moreover, it should be taken into account that the presentation and symptomatology of the eating disorders (subtype of AN or BN) is probably not only dependent on stable factors such as personality, but also changes in personality functioning due to other situational factors that require different coping behavior. Thus, the frequent transition of patients between subtypes and, in some cases, between disorders would be more explainable (23–25). The DSM-5 also recommends using the subtype of AN as a description of current rather than long-term symptomatology (26). Purging behavior could be a coping strategy used more often in stressful parts of live requiring a high level of emotion regulation. As suggested by the results of a therapy study published in 2017, improvement in emotion regulation predicts levels of purging behavior (27). In the latest version of the OPD {OPD-3; [OPD (28)]}, the personality structure is not regarded as an exclusively rigid characteristic. Among other things, this takes into account, mentalization research, which also describes a situational dependence of mentalization abilities (29). Based on these thoughts, the current subtype of anorexia nervosa may allow conclusions about the level of current personality functioning. However, to validate this connection, further research is definitely needed.

In a recent pilot study (30) based on secondary data of *N* = 60 ED patients, we explored potential differences in personality functioning between patients with AN-R, AN-P, and BN as measured with the OPD personality structure axis. On almost all dimensions, patients with AN-R showed the best personality functioning compared with AN-P and BN patients. These differences were most pronounced for the self-perception and self-regulation dimensions. There was a tendency for differences on other dimensions such as regulation of self-esteem that did not reach statistical significance. We concluded that if the findings could be confirmed that this evidence may be used to improve treatment stratification for ED patients.

The aim of the present study was to provide a deeper insight into differences in personality functioning across the three groups of patients with EDs, to confirm the previous findings (30) in a larger sample using a prospective design and to extend them by exploring concealed links between features of eating disorders and impairments in personality functions. Recent evidence from an intervention study in patients with purging behavior supports the assumption that these patients may have more difficulties in regulating their emotions because they benefited rapidly from an intervention that focused on these abilities (27). These findings, together with our previous results led to three specific hypotheses including that (1) patients with purging behavior (AN-P and BN) have a more disintegrated personality structure than patients with AN-R; that (2) patients with AN-R have a higher capacity of affect tolerance than both AN-P and BN subjects; and that (3) patients with higher scores in overall eating pathology show a more disintegrated OPD personality structure.

## 2. Materials and methods

### 2.1. Study design, sample, and procedure

The study was designed as a prospective multicenter study. The study center was located at the Department of Psychosomatic Medicine, Charité - Universitätsmedizin Berlin. Patients undergoing inpatient treatment for their eating disorders were recruited from three clinics for psychosomatic medicine in Berlin between 07/2018 and 11/2020 including Charité–Universitätsmedizin Berlin, Kliniken im Theodor-Wenzel-Werk (TWW) and Gemeinschaftskrankenhaus Havelhöhe (GKH). Patients with a clinical diagnosis (or a suspected diagnosis) of any eating disorder were asked to participate during their first week of treatment. Following informed consent, patients were administered a comprehensive battery of tests that included several psychometric instruments assessing eating disorder and personality functioning. Patients could decide whether they wanted to participate via paper-and-pencil or electronic assessment using their own tablet/smartphone, an in-hospital computer (only at GKH) or a tablet provided by the study personnel (only at Charité). The study was approved by the institutional review board at Charité – Universitätsmedizin Berlin (EA4/011/18) and was conducted in accordance with the Declaration of Helsinki.

### 2.2. Instruments

#### 2.2.1. Munich ED-Quest: the Munich eating and feeding disorder questionnaire

This self-report questionnaire was developed as a comprehensive assessment of eating disorder symptoms and for deriving eating disorder diagnoses according to DSM-5 and ICD-10. It consists of 65 items which are primarily rated on a five-point scale between 0 “no at all” and 4 “very severely.” In a slightly different wording, the items include all criteria for DSM-5 and ICD-10 ED diagnoses of AN, BN, binge-eating disorder (BED), rumination disorder, avoidant/restrictive food intake disorder, and night-eating disorder (NES). Furthermore, it measures a wide range of disordered eating behavior and cognitions beyond the criteria of the classification systems. Three subscales cover “preoccupation with figure and weight,” “bingeing and vomiting,” and “inappropriate compensatory behavior.” A validation study shows satisfying psychometric properties including reliability (Cronbach’s α = 0.96), test-retest reliabilities for the three subscales between α = 0.95 and α = 0.98 and construct validity, convergent validity with three entrenched eating disorder questionnaires: SIAB-S (Structured Inventory for Anorexic and Bulimic Eating Disorders), EDI-2 (Eating Disorder Inventory), and EDE-Q (Eating Disorder Examination-Questionnaire) (31). Besides that, applying the diagnostic algorithm for DSM-5 showed a specificity of 0.980 for AN and 0.974 for BN validated by expert diagnoses (31, 32).

#### 2.2.2. EDE-Q: eating disorder examination-questionnaire

To facilitate the international comparability, eating disorder symptoms were also measured with the EDE-Q, a well-established self-report questionnaire based on the Eating Disorder Examination (EDE) Interview (33, 34). Compared to the Munich ED-Quest this self-report instrument was not built to support diagnostics but to quantify eating pathology. It consists of 28 items using a 7-point rating scale. Four subscales reflect “Restraint,” “Eating Concern,” “Weight Concern,” and “Shape Concern.” A global eating pathology score can be derived as the average of the four subscale scores. High correlations between the EDE-Q and the EDE were reported in several studies in clinical and community samples (35, 36). The instrument shows satisfying internal consistency with Cronbach’s alpha coefficients ranging from α = 0.70 to α = 0.83 in a clinical sample (37) and α = 0.83 in our sample.

#### 2.2.3. OPD-SQ: operationalized psychodynamic diagnosis structure-questionnaire

The OPD-SQ is a self-report instrument assessing personality structure as defined by the OPD-System (Levels of Structural Integration Axis, LSIA). It consists of 95 items which are rated on a 5-point Likert scale from “no agreement at all” to “very high agreement.” Eight scales and 21 subscales reflect a wide range of aspects of personality functioning that relate to the self and the object (see Figure 1 for an overview). A total score, which can be calculated as the average score of the 21 subscales, is an indicator of the overall structural level of functioning, where higher scores indicate greater structural impairment. Thus, the OPD-SQ gives an overview on the overall structural integration (global scale), the four basic functions and their differentiations in relation to the self and others (8 scales) and 21 of the 24 subdimensions (21 subscales).

The instrument demonstrates sufficient internal consistency reliability: In our sample it showed a Cronbach’s α of 0.96 for the overall scale. Several validation studies found satisfying psychometric properties. The accordance with expert-ratings of the LSIA (38, 39), as well as with other measures of personality and attachment (38), and the number of DSM-IV PD diagnoses (40), was high. In addition to the full version of the OPD-SQ, results were also analyzed based on a 12-item-shortform (OPD-SQS) which was recently introduced (41, 42). The OPD-SQS has a three-factor structure including self-perception, establishing contact, and relationship model.

#### 2.2.4. PID-5-BF: the personality inventory for DSM-5–brief form

The PID-5-BF is a 25-item self-report personality trait assessment which is rated on a 4-point scale from “very false or often false” to “very true or often true.” It is a brief version of the Personality Inventory for DSM-5 (PID-5) that includes 220 items. The PID-5-BF includes the five domains “negative affect,” “detachment,” “antagonism,” “disinhibition,” and “psychoticism.” The mean of all items is used as total score of overall personality dysfunction. The reliability of the PID-5-BF was found to be sufficient. Cronbach’s alpha for the PID-5-BF total score was 0.83, test-retest reliability of the subscales ranged from *r* = 0.78 to *r* = 0.97 (43). In our sample the total score showed a Cronbach’s alpha of 0.87. Findings on convergent and discriminant validity also support the value of the PID-5-BF (44, 45).

#### 2.2.5. BFI-10: big five inventory-10

This measurement assesses the Big Five personality traits (Five Factor Model, FFM) “Neuroticism,” “Extraversion,” “Openness,” “Agreeableness,” and “Conscientiousness” using 10 items. The items are scored on a 5-point Likert scale ranging from “disagree strongly” to “agree strongly.” The BFI-10 was developed based on the 44-item Big Five Inventory (BFI-44) (46). High correlations with other instruments measuring the Big Five and peer ratings suggest acceptable validity, test-retest correlations as well as a Cronbach’s alpha between α = 0.74 and α = 0.89 pointed at sufficient reliability (47). In our sample it showed an α = 0.75.

#### 2.2.6. PROMIS-29

The PROMIS-29 is a comprehensive instrument from the Patient-Reported Outcome Measurement Information System (PROMIS^®^) that measures eight domains: depression, anxiety, physical function, pain intensity, pain interference, fatigue, sleep disturbance, and ability to participate in social roles and activities. The eight domains cover the most relevant areas of self-reported health for the vast majority of individuals with chronic conditions. Although a total score can be derived, in this study we use the scores that reflect the individual PROMIS domains. PROMIS domains are based on item-response-theory measurement models that allow to determine the scores on the latent domain traits of individuals with high precision. A score of 50 represents the average of the general population. A score of 60 or 40, for example, means that the persons’ score is one standard deviation above or below the average of the reference population (standard deviation = 10). Lower values correspond to better health, except for the physical function domain, where lower values reflect lower physical functioning (48).

### 2.3. Statistical analyses

All descriptive und inferential analyses were carried out with IBM SPSS Statistics Version 24 (IBM corp., Armonk, NY, USA). All effect sizes were converted into Cohen’s d for comparability and interpreted according to Sawilowsky (49) as *d* > 0.2 = small, *d* > 0.5 = medium, *d* > 0.8 = large and *d* > 1.2 = very large. To find an appropriate sample size for the analysis of differences on the main scales of the OPD-SQ, we performed a sample size calculation (calculated with G*Power version 3.1). Based on the results of the pilot study, we expected large effect sizes so we calculated *a priori* a MANOVA with f^2^(Pillai’s trace; V) = 0.14 and *p* < 0.05, which resulted in a sample size of 108 patients (50).

Cases were divided into three groups based on the ED-Quest responses. Using a validated algorithm provided by the ED-Quest authors, patients were categorized into anorexia nervosa, bulimia nervosa, typical, and atypical cases according to the ICD-10 classification. ED groups included AN-R, AN-P, and BN.

#### 2.3.1. Identification of differences in personality functioning

First, potential confounders of the group comparisons were identified by comparing the groups in terms of their sociodemographic variables (age, sex, education level, living situation, employment, and marital status), the study center (Charité, TWW, and GKH), their BMI, and clinical characteristics (using PROMIS domains). To achieve this goal, a series of analysis of variance tests (ANOVAs) were performed. We decided not to include BMI as a covariate in our primary analysis as it was not associated with the dependent variables (OPD main scales and subscales) but with the independent variable (ED group) and could therefore have resulted in a reduction in group differences.

In the next step, the three groups were compared in terms of their personality structure/functioning/disorder levels. To compare the domains and subscales of personality measures (OPD-SQ, PID-5-BF, and BFI-10) across the three groups (AN-R, AN-P, and BN) multivariate analyses of covariance (MANCOVAs) were used to enable the inclusion of confounding variables. Analysis of variance tests with covariates (ANCOVA) and without covariates (ANOVA) were used to compare total scores of personality (functioning) measures across ED groups. Bonferroni corrections were used to control for the family-wise error rate. To evaluate the sensitivity of the multivariate analyses, *post hoc* power analyses were performed.

#### 2.3.2. Associations between eating disorder symptoms and personality functioning

To identify individual eating disorder symptoms that were most likely to be associated with dysfunction in personality structure, we used a two-step method. First, we calculated correlations between eating disorder symptoms and the personality functioning. Therefore, we used the items of the Munich ED-Quest and the EDE-Q and correlated them with the global scale and each main- and subscale of the OPD-SQ.

Second, we estimated regression models for each OPD-SQ main- and subscale and for the global scale using all items of the Munich ED-Quest and the EDE-Q as predictors. A step-wise multiple regression procedure in SPSS was used to determine the five items that best predicted personality structure.

## 3. Results

### 3.1. Sample

*N* = 110 patients with complete datasets were included in the analyses. A total of 13 patients had to be excluded because required questionnaires such as the OPD-SQ or the ED-Quest were not completed. About two thirds of the included cases were treated at Charité (*n* = 71) and about one third at the other study centers (TWW, *n* = 24; GKH *n* = 15). Sample characteristics are shown in Table 1. Whereas 28 cases met the criteria of an AN-R, 40 cases were identified as AN-P and 42 cases met the criteria for BN. The mean age was 30.8 years (*SD* = 11.38), the majority of the participants were female *n* = 99 (90.0%) and *n* = 43 (39.0%) participants lived alone. The comparison of the demographic variables across the ED groups (AN-R, AN-P, and BN) showed no significant differences except for BMI and living situation (*p* < 0.05). BMI was significantly lower in the AN-R and AN-P groups compared to the BN group; patients with AN-R were more likely to live with their family than BN or AN-P patients. As expected, furthermore the BMI was significantly lower in AN-R and AN-P patients than in BN patients.

**TABLE 1:** Sample characteristics.

	AN-R	AN-P	BN	
	*n* = 28	*n* = 40	*n* = 42	*p*
Age in years M (SD)	33 (15.45)	33 (12.05)	31 (10.12)	0.69
Range in years	18−64	18−61	19−53	
Female gender *N* (%)	25 (89)	37 (93)	37 (88)	0.44
Living situation *N* (%)				0.28
Single	10 (36)	18 (45)	15 (36)	
With parents/partner/living community	18 (64)	22 (55)	27 (64)	
Educational level *N* (%)				0.27
University entrance diploma or higher	7 (25)	11 (28)	9 (21)	
Certificate of secondary education	13 (46)	8 (20)	17 (40)	
Certificate of primary or lower secondary education	8 (29)	20 (50)	14 (33)	
Without	0 (0)	1 (3)	2 (5)	
BMI in kg/m^2^ M (SD)	17.4 (4.32)	18.1 (3.92)	23.9 (7.39)	<0.001
x̃ in kg/m^2^	16.5	17.0	21.6	
Range of BMI	11.5−24.6	11.2−24.9	17.6−50.1	
Study center *N* (%)				0.12
Charité	18 (82)	25 (68)	28 (67)	
TWW	3 (14)	10 (27)	11 (26)	
GKH	1 (5)	2 (5)	3 (7)	

AN-R, restricting type anorexia nervosa; AN-P, purging type anorexia nervosa; Charité, Charité – Universitätsmedizin Berlin; TWW, Kliniken im Theodor-Wenzel-Werk; GKH, Gemeinschaftskrankenhaus Havelhöhe; BMI, body mass index; BN, bulimia nervosa; M, mean; N, number; *p*, *p*-value; statistical significance, SD, standard deviation; x̃, median.

### 3.2. Personality and personality structure–differences between eating disorder types

#### 3.2.1. Personality structure across eating disorder groups (OPD-SQ, OPD-SQS)

**FIGURE 2:**
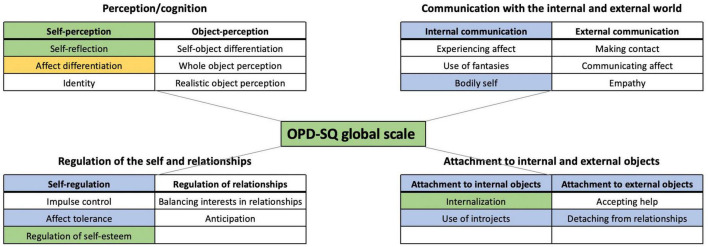
Significant differences between the three eating disorder groups on the main- and subscales of the OPD-SQ. Significant differences MANOVA’s (α = 0.05): Blue: AN-R < AN-P/BN; Green: AN-R < BN; Yellow: AN-R/AN-P < BN; (lower values indicate a better integrated structural level).

The (M)ANOVA models used to investigate whether the OPD main- and subscales showed significant differences between the three groups of EDs displayed medium to very large effects (Subscales: *F* = 1.459, *p* = 0.048, *d* = 1.179; Main scales: *F* = 2.294, *p* = 0.027, *d* = 0.853; global scale: *F* = 5.3486, *p* = 0.005, *d* = 0.64). The analyses revealed significant differences on eight subscales, on five main scales and on the global scale. The latter showed significant differences between AN-R and BN patients, whereas AN-P patients were not different from either group (Figure 2 for an overview and Table 2 for statistic values). Similar to the OPD-SQ, we found differences between the global scales and subscales of the short instrument version (i.e., OPD-SQS). We continue with describing significant *post hoc* differences between the three ED groups in detail.

**TABLE 2:** Differences between eating disorder groups in personality structure (OPD-SQ) and other personality models (MANOVA).

Model	Statistics	*F*	*p*	η^2^	Cohen’s *d*	
**OPD-SQ**						
Global scale	3.558	5.486	**0.005**	0.093	0.640	AN-R < BN
Main scales	0.182	2.294	**0.027**	0.154	0.853	Figure 2
Self-perception	4.907	6.469	**0.002**	0.108	0.696	Figure 2
Object perception	1.247	2.336	0.102			
Self-regulation	2.663	5.249	**0.007**	0.089	0.625	Figure 2
Regulation of relationships	1.134	1.619	0.203			
Internal communication	1.550	3.963	**0.022**	0.069	0.545	
External communication	0.002	0.006	0.994			
Attachment to internal objects	3.380	5.770	**0.004**	0.097	0.656	Figure 2
Attachment to external objects	3.045	5.827	**0.004**	0.098	0.659	Figure 2
Subscales	0.517	1.459	**0.048**	0.258	1.179	Figure 2
Self-reflection	2.670	2.685	0.073			
Affect differentiation	6.582	8.303	**0.001**	0.134	0.787	Figure 2
Identity	6.357	6.362	**0.002**	0.106	0.689	Figure 2
Self–object differentiation	1.048	1.644	0.198			
Whole object perception	2.920	3.231	**0.043**	0.057	0.492	Figure 2
Realistic object perception	0.546	0.657	0.520			
Impulse control	2.096	2.137	0.123			
Affect tolerance	4.433	4.641	**0.012**	0.080	0.59	Figure 2
Regulation of self-esteem	1.849	3.161	**0.046**	0.056	0.487	Figure 2
Balancing interests	1.654	1.941	0.149			
Anticipation	1.023	1.336	0.267			
Experiencing affect	2.505	3.167	**0.046**	0.056	0.487	Figure 2
Use of fantasies	0.069	0.079	0.924			
Bodily self	6.549	6.645	**0.002**	0.110	0.703	Figure 2
Making contact	0.499	0.420	0.658			
Communicating affect	1.040	1.574	0.212			
Empathy	1.684	1.928	0.150			
Internalization	3.735	3.602	**0.031**	0.063	0.519	Figure 2
Use of introjects	3.105	4.735	**0.011**	0.081	0.594	Figure 2
Accepting help	1.278	1.407	0.249			
Detaching from relationships	6.705	7.234	**0.001**	0.119	0.735	Figure 2
**OPD-SQS**						
Total score	2.525	4.229	**0.017**	0.073	0.561	AN-R < BN
Self-perception	5.338	5.420	**0.006**	0.092	0.637	AN-R < BN
Establishing contact	0.333	0.484	0.618			
Relationship model	3.791	3.762	**0.026**	0.066	0.532	AN-R < BN
**PID-5-BF**						
Total score	0.670	3.492	**0.034**	0.063	0.519	AN-R < BN
Negative affect	0.701	1.937	0.149			
Detachment	0.911	1.982	0.143			
Antagonism	0.386	1.541	0.219			
Disinhibition	1.085	2.512	0.086			
Psychoticism	0.503	1.101	0.336			
**BFI**						
Extraversion	1.237	1.126	0.328			
Neuroticism	1.770	2.197	0.116			
Openness	0.331	0.294	0.746			
Conscientious- ness	3.848	4.554	**0.013**	0.079	0.586	BN < AN-R/AN-P
Agreeableness	2.214	3.055	0.051			

Mainscales of the OPD-SQ are indicated in bold. AN-R, anorexia nervosa restricting type; AN-P, anorexia nervosa purging-type; BN, bulimia nervosa; η^2^, partial eta square; *F*, multivariate *F*-statistics; *p*, significance (Bonferroni corrected); MANOVA, multivariate analysis of variance; OPD-SQ, OPD structure questionnaire; BFI, big-five Inventory; PID-5-BF, PID-5 brief Inventory.

##### 3.2.1.1. Differences between AN-R and both AN-P and BN

On several OPD scales, the AN-R group differed significantly from the two other ED groups that are associated with purging behavior (AN-P and BN) (see Figure 2). These included the main scale “self-regulation” (*F* = 5.249, *p* = 0.007, *d* = 0.625) and the corresponding subscale “affect tolerance” (*F* = 4.641, *p* = 0.012, *d* = 0.590), as well as the main scale “attachment to internal objects” (*F* = 5.770, *p* = 0.004, *d* = 0.656) and its subscale “use of introjects” (*F* = 4.735, *p* = 0.011, *d* = 0.594), and the main scale “attachment to external objects” (*F* = 5.827, *p* = 0.004, *d* = 0.659) with its subscale “detaching from relationships” (*F* = 7.234, *p* = 0.001, *d* = 0.735). Furthermore, there was a significant difference at the main scale “internal communication” (*F* = 3.963, *p* = 0.022, *d* = 0.545) and its subscale “bodily self” (*F* = 6.645, *p* = 0.002, *d* = 0.703).

##### 3.2.1.2. Differences between AN-R and BN

The analyses also revealed many significant differences of the OPD-SQ main scales and subscales between AN-R and BN only. BN patients showed a greater impairment (up to severe/extreme) than patients with AN-R, whereas the average scores of the AN-P group turned out to be between both, AN-R and BN and thus did not differ significantly from the other groups. The main scale “self-perception” (*F* = 6.469, *p* = 0.002, *d* = 0.696) and the corresponding subscale “identity” (*F* = 6.362, *p* = 0.002, *d* = 0.689) demonstrated differences between AN-R and BN. As opposed to the domains which they belong to, the subscales “regulation of self-esteem” (*F* = 3.161, *p* = 0.046, *d* = 0.487) and “internalization” (*F* = 3.602, *p* = 0.031, *d* = 0.640) did also show differences between AN-R and BN.

Furthermore, the OPD-SQS scales self-perception (*F* = 5.420, *p* = 0.006, *d* = 0.637) and relationship model (*F* = 3.762, *p* = 0.026, *d* = 0.532) showed differences between patients with AN-R and BN.

##### 3.2.1.3. Differences between BN and the two AN groups

We found a significant difference on OPD-SQ main- and subscales between patients with BN and both AN groups only on the subscale “affect differentiation” (*F* = 8.303, *p* < 0.001, *d* = 0.787) with BN showing significantly greater impairment than the AN-R and AN-P.

#### 3.2.2. Big five personality traits and DSM-5 personality functioning across eating disorder groups (PID-5, BFI-10)

The total score of overall personality dysfunction of the PID-5-BF differed significantly (*F* = 3.492, *p* = 0.034, *d* = 0.519). *Post hoc* analyses showed that patients with BN had a significant higher mean score (i.e., more pronounced personality dysfunction) than patients with AN-R (*t* = 2.501, *p* = 0.014, *d* = 0.491). We did not observe differences in any of the five domains of the PID-5 across the three ED groups (Table 2).

When using the scales of the big five BFI-10 as independent variables, the analysis showed a significant difference between the ED groups only on the scale “agreeableness” (*F* = 3.120, *p* = 0.012, *d* = 0.780). Pairwise comparisons indicated that AN-R patients had a significantly higher mean score on the domains “agreeableness” (*t* = 2.470, *p* = 0.015, *d* = 0.478) compared to BN patients.

### 3.3. Features of EDs and their association with personality structure

Table 3 shows the Pearson correlations between the OPD-SQ (subscales and global scale)/OPD-SQS (subscales and global scale) and the Munich ED-Quest/EDE-Q scores. Many significant correlations were found, some with large effects (*r* > 0.5).

**TABLE 3:** Correlations of the OPD-SQ and -SQS scales with eating pathology scores.

	Munich ED-Quest					EDE-Q				
OPD-SQ		Preoccupation with figure and weight	Bingeing and vomiting	Inappropriate comp. behavior	Total score	Restraint	Eating concern	Weight concern	Shape concern	Total score
Self-perception	*r*	0.486***	0.443***	0.360***	0.570***	0.325**	0.431***	0.402***	0.468***	0.472***
Self-reflection	*r*	0.429***	0.320**	0.349***	0.481***	0.301**	0.405***	0.338***	0.466***	0.431***
Affect differentiation	*r*	0.468***	0.453***	0.321**	0.555***	0.311**	0.398***	0.399***	0.452***	0.449***
Identity	*r*	0.432***	0.439***	0.314**	0.523***	0.279**	0.378***	0.364***	0.367***	0.415***
Object perception differentiation	*r*	0.500***	0.320**	0.446***	0.547***	0.314**	0.307**	0.286**	0.360***	0.365***
Self–object differentiation perception	*r*	0.523***	0.303**	0.369***	0.541***	0.340***	0.370***	0.356***	0.420***	0.423***
Whole object perception	*r*	0.393***	0.211*	0.375***	0.418***	0.297**	0.244*	0.270**	0.344***	0.306**
Realistic object perception	*r*	0.339***	0.288***	0.363***	0.411***	0.151	0.164	0.100	0.144	0.197*
Self-regulation	*r*	0.484***	0.362***	0.366***	0.538***	0.356***	0.423***	0.374***	0.448***	0.459***
Impulse control	*r*	0.245**	0.28**	0.258**	0.324***	0.185	0.192*	0.157	0.203*	0.2*
Affect tolerance	*r*	0.493***	0.33***	0.4***	0.538***	0.344***	0.414***	0.345***	0.388***	0.425***
Regulation of self-esteem	*r*	0.424***	0.243*	0.192*	0.417***	0.331***	0.42***	0.414***	0.512***	0.5***
Regulation of relationships	*r*	0.454***	0.295**	0.372***	0.493***	0.267**	0.287**	0.261***	0.312***	0.317***
Balancing interests	*r*	0.431**	0.302**	0.342**	0.475**	0.289**	0.219*	0.225*	0.247**	0.272**
Anticipation	*r*	0.414***	0.245**	0.351***	0.442***	0.205*	0.318***	0.261**	0.337***	0.32***
Internal communication	*r*	0.331***	0.347***	0.234**	0.404***	0.307***	0.247**	0.36***	0.509***	0.404***
Experiencing affect	*r*	0.33***	0.346***	0.263**	0.409***	0.257**	0.163	0.244**	0.32***	0.282**
Use of fantasies	*r*	-0.182	-0.056	-0.182	-0.179	-0.08	-0.135	-0.054	0.041	-0.073
Bodily self	*r*	0.486***	0.39***	0.365***	0.55***	0.412***	0.435***	0.505***	0.629***	0.563***
External communication	*r*	0.16	0.011	0.084	0.129	0.106	0.015	0.031	0.06	0.07
Making contact	*r*	0.161	0.047	0.148	0.155	0.129	0.078	0.069	0.129	0.165
Communicating affect	*r*	0.386***	0.221*	0.273**	0.399***	0.232*	0.244*	0.265**	0.286**	0.291**
Empathy	*r*	-0.238*	-0.226*	-0.259**	-0.296***	-0.161	-0.276**	-0.25**	-0.288***	-0.317***
Attachment to internal objects	*r*	0.565***	0.343***	0.394***	0.592***	0.417***	0.432***	0.456***	0.521***	0.542***
Internalization	*r*	0.461***	0.325***	0.357***	0.508***	0.307***	0.324***	0.322***	0.379***	0.408***
Use of introjects	*r*	0.503***	0.249***	0.305***	0.495***	0.416***	0.42***	0.47***	0.524***	0.527***
Attachment to external objects	*r*	0.532***	0.325***	0.282**	0.54***	0.399***	0.401***	0.397***	0.48***	0.495***
Accepting help	*r*	0.362***	0.143	0.073	0.315***	0.256**	0.237*	0.214*	0.301**	0.282**
Detaching from relationships	*r*	0.448***	0.348***	0.35***	0.505***	0.349***	0.368***	0.386***	0.431***	0.467***
Global scale	*r*	0.571***	0.402***	0.415***	0.622***	0.403***	0.42***	0.419***	0.512***	0.509***
**OPD-SQS**										
Total score	*r*	0.523***	0.398***	0.381***	0.583***	0.326***	0.394***	0.357***	0.427***	0.464***
Self-perception	*r*	0.439***	0.392***	0.38***	0.522***	0.278***	0.385***	0.349***	0.39***	0.43***
Establishing contact	*r*	0.323***	0.214*	0.254***	0.351***	0.198**	0.24*	0.162	0.266**	0.289**
Relationship model	*r*	0.514***	0.358***	0.299**	0.546***	0.317***	0.333***	0.352***	0.385***	0.417***

r = Pearson correlation; *p < 0.05; **p < 0.01; ***p < 0.001.

For example, attachment to internal (*r* = 0.565) and external objects (*r* = 0.532) correlates strongly with “Preoccupation with Figure and Weight”. The degree of development of the bodily self correlates strongly with all scales of the EDE-Q, especially with the Shape Concern scale (*r* = 0.629). The overall eating pathology of the Munich ED-Quest shows a strong correlation with the global scale of the OPD-SQ (*r* = 0.622). Some of the dimensions of the OPD-SQS also correlated strongly with specific eating disorder pathologies. For example, the relationship dimension correlated strongly with “Preoccupation with Figure and Weight” as measured by the Munich ED-Quest (*r* = 0.514).

We calculated one multiple regression model for each main scale of the OPD-SQ and one for the global scale. To improve readability, we only report significant models together with the best predictor of this model below.

Using the OPD-SQ global scale as dependent variable the five best predicting items of the Munich ED-Quest and the EDE-Q explained almost 40% of the variance of global personality structure (adjusted *R*^2^ = 0.389; *F* = 14.859; *p* < 0.001). The magnitude of the association and item content of these five items are provided in Supplementary Table 1. The total score of the Munich ED-Quest significantly predicted global personality structure as measured by OPD-SQ (stand. β = 0.650; *p* < 0.001)–more eating disorder symptoms predicted a more disintegrated structure. This score also was the best predictor of the OPD-SQ scale “self-regulation” (stand. β = 0.449; *p* < 0.001). The whole model predicted almost 30% of the variance (adjusted *R*^2^ = 0.281; *F* = 9.527; *p* < 0.001).

Regression models predicting OPD-SQ scales indicated that symptoms of EDs could predict scores of four main scales and that each of these four models explained more than 30% of the variance in OPD-SQ scale scores (Supplementary Table 1): 34.3% of the variance of the main scale “self-perception” was explained (adjusted *R*^2^ = 0.343; *F* = 12.379; *p* < 0.001) by the item on consumption of high-calorie foods during binge eating (stand. β = 0.489; *p* < 0.001). This ED feature was also the best predictor of the OPD-SQ scale “attachment to external objects” (stand. β = 0.377; *p* = 0.032). This model predicted 34% of the available variance (adjusted *R*^2^ = 0.340; *F* = 11.630; *p* < 0.001). It predicted about one third of the variance of “object-perception” (adjusted *R*^2^ = 0.337; *F* = 11.973; *p* < 0.001). The feeling of a loss of control of one’s life predicted “Object-perception’ with the highest share of variance in this model (stand. β = 0.499; *p* < 0.001). Furthermore, 31.1% of the variance of “Internal communication” was predicted by the model (adjusted *R*^2^ = 0.311; *F* = 10.275; *p* < 0.001), the best predictor of ‘internal communication’ was the EDE-Q scale “shape concern” (stand. β = 0.390; *p* = 0.001).

## 4. Discussion

In this prospective, cross-sectional multicenter study we describe differences and similarities in personality functioning across different types of eating disorders, using two-dimensional personality functioning instruments. We were able to largely confirm our findings from a recent pilot study on differences of personality functioning according to the OPD structural axis (30). In summary, there is evidence that capabilities and limitations on several personality functioning facets are more similar between AN-P and BN patients than between AN-P and AN-R patients, although current treatment guidelines suggest treating AN-P and AN-R patients similarly and using a different approach for BN patients (51). In times of increasing relevance of individualized treatment programs these findings suggest that personality functioning facets may be meaningful indicators for treatment stratification in patients with eating disorders.

We were able to confirm most of our hypotheses. AN-R patients demonstrated better overall personality functioning according to OPD than patients with BN, which confirmed Hypothesis 1 in part. Other than expected the difference between AN-R and AN-P patients did not reach statistical significance as was the case in our pilot study. Note, however, that the difference between AN-R and AN-P patients closely approaches the significance threshold of *p* ≤ 0.05 and that we used Bonferroni corrections to adjust for multiple comparisons throughout the manuscript to maintain a high level of scientific rigor. If a less restrictive method such as the Tukey method (52) had been used the significance would have exceeded the threshold of *p* ≤ 0.05. In contrast to this close-to-significant difference between AN-R and AN-P, values of AN-P and BN patients were very similar and there was no tendency toward a significant difference between those groups. The results of overall personality functioning according to DSM-5 (PID-5) did show a similar pattern, although less pronounced. Whereas, however, AN-R and BN differed significantly in their levels of personality functioning, the difference between AN-R and AN-P did not reach statistical significance, not even if the Tukey method was used to adjust for multiple comparisons.

In addition to differences of overall personality functioning across ED groups findings indicate several differences on the main scales and subscales of the OPD-SQ which support Hypothesis 1. Four out of eight main scales (i.e., internal communication, self-regulation, attachment to internal objects, and attachment to external objects) indicated differences in personality functioning facets between AN-R and both AN-P and BN. Only on the subscale affect differentiation both AN groups resulted in better personality functioning than the BN group. That means that in the population under investigation AN-P patients have a similar capability as AN-R patients to distinguish different emotions from each other and that BN patients are less capable of doing so. This finding was surprising, given that affect differentiation was similar for both BN and AN-P patients and higher than for AN-R patients in the pilot study (30). Further evidence on this dimension is warranted, however, if the results of the present study are confirmed this personality functioning facet could prove useful as an indicator for differentiating between BN and AN-P patients.

The vast majority of differences across ED groups relate to “internal” functions, which reflect capabilities of the self, including self-perception, internal communication, self-regulation, and attachment to internal objects as opposed to “external” functions that reflect capabilities of communication and interaction with others (Figure 2; Table 3). This is not surprising given that differences in common symptoms of the ED groups pertain to behaviors that are used for regulating affect such as self-induced vomiting, misusing of laxatives or medications, or exercising excessively. Among the internal functions that demonstrated higher levels in AN-R patients compared to BN and AN-P patients was affect tolerance (Hypothesis 2). Growing evidence has related unsuccessful regulation of emotional states–which includes the ability to tolerate (strong) emotions–to impulsive behaviors in eating disorders (53–55). The findings suggest that both AN-P and BN patients–who tend to have more difficulties in tolerating emotions–might particularly benefit from treatment approaches that focus on these difficulties. In addition to common approaches such as skills training (56, 57) specific treatment programs are available that help patients challenge the basis of their emotional distress (58).

Other than expected the association between more pronounced overall eating pathology and lower personality functioning was weak (Hypothesis 3). Admittedly, the correlations between the OPD and PID total scores as well as the EDEQ and ED-Quest total scores were moderate (r between 0.2 and 0.6). However, previous investigations of personality functioning instruments have indicated that these instruments also capture symptom-level severity, and this was found to be especially the case for the OPD-SQ (39, 42), which is why we also calculated partial correlations that accounted for symptom severity such as depression, anxiety, or fatigue. When we controlled for these variables we did not find an association between eating disorder severity and DSM-5 personality functioning (PID), and we did only find a weak correlation between eating disorder severity and OPD personality functioning (OPD-SQ). One has to note, however, that this approach only investigates linear relationships although the (categorical) classification suggests a non-linear relationship. Another explanation is that there is just no (strong) association between personality functioning and eating pathology. That means that there may be patients with similar eating disorder burden that have different levels of personality functioning.

We think that our findings might have some implications for clinical practice and future research. Given the marked differences in personality functioning facets between the two subtypes of anorexia nervosa a revision of the classification may be considered that takes more into account the differences between the two AN types. For example, the AN types may be regarded as separate diagnostic entities. That may have desirable effects on research, in particular on the differential investigation of treatment effects in AN subtypes. Previous clinical trials that investigated the efficacy of AN treatments did usually include both subtypes in the same intervention group and some of those studies did not even report the subtype as part of the sample description (59). This is surprising given the fact that there is some evidence that the AN subtype or binge-/purging-behavior are moderators or mediators of treatment outcomes (60, 61). For example, Le Grange et al. (61) reported that the effect of family therapy was larger for AN-P patients than for AN-R patients.

The primary goal of AN treatments remains weight gain, followed by the reduction of other eating disorder symptomatology such as body-image disturbances (62), while increasing personality functioning capabilities (or structural capabilities according to OPD) such as on emotion regulation is usually not a primary treatment target. However, improving emotional self-regulation belongs to the key focuses of psychotherapy in bulimia nervosa, for example as part of modified dialectical behavioral therapy (63), interpersonal therapy (64), or psychodynamic psychotherapy (64). Naturally, because of the imminent complications, weight gain is an important part of AN management. It might be speculated, however, that addressing personality functioning capabilities in the treatment of AN-P patients might be beneficial for the outcome.

Even though there is no psychotherapy approach that has proven to be superior in AN, guideline-recommended kinds of psychotherapies are available (i.e., MANTRA and FPT) for which good results in the treatment of anorexia nervosa in adults have been demonstrated in recent years (65). Because some of these therapies address facets of personality functioning/structure, it might be useful to select the therapy according to these facets and thus individualize it by the choice of the therapy method. Since the valid assessment of personality functioning takes time, an economical alternative would be the assignment based on the subtype of anorexia.

In addition, a more individualized therapy, initially oriented to the subtype of the eating disorder, is conceivable. A similar concept, which is also oriented to personality functioning, has already been proposed for the treatment of depressive patients (66). In patients who currently have a diagnosis of BN, affect differentiation should be increasingly addressed. If patients currently suffer from purging symptoms, it would make sense to strengthen emotion regulation (as described above) and to focus on the relationship with the therapist (e.g., using transference focused psychotherapy) in order to address vulnerabilities in attachment skills.

### 4.1. Limitations

Some limitations have to be acknowledged. First, although the sample size is larger than in our pilot study (30), it is still relatively small. If the sample had been larger, some of the differences that were close to the predefined significance threshold may have become significant. Second, two out of three recruiting sites did only contribute a minority of patients which may have led to bias (e.g., selection bias). Third, comparison of personality functioning levels with other groups of eating disorders such as with binge-eating disorders would have been desirable. However, this was not possible due to the nature of the clinical population in the clinics. In addition, cases with atypical AN were included, and the classification into the three groups was not based on an expert interview but on a validated self-report questionnaire. Last, the instrument used in this study (OPD-SQ) is based on the OPD system which is primarily applied in German-speaking countries. Although there has been growing international recognition in recent years (67, 68), it remains unclear whether this system will be widely used internationally.

## 5. Conclusion

We found that on average personality functioning in AN-P and BN patients was lower than in AN-R patients. These findings suggest that BN and AN-P patients might benefit from an approach that aims at improving capabilities on various personality facets. Furthermore, our findings indicate that patients with different subtypes of AN should probably be investigated separately in clinical trials. This means that studies should either focus on one AN subgroup, which would limit the sample size, or studies should calculate their sample size to facilitate *post hoc* subgroup analyses. This would allow to examine more closely more specialized interventions.

## Data availability statement

The original contributions presented in this study are included in the article/Supplementary material, further inquiries can be directed to the corresponding author.

## Ethics statement

This study was approved by the Institutional Ethics Committee of the Charité – Universitätsmedizin Berlin (protocol number: EA1/114/10). The patients/participants provided their written informed consent to participate in this study and all investigations in the present study were conducted in accordance with the Declaration of Helsinki.

## Author contributions

JR, AO, BV, and MR contributed to the design of the study. JR, AO, C-SK, and SB-M were responsible for data assessment. JR organized the database, performed the statistical analysis, and wrote the draft of the manuscript. TH and AO wrote sections of the manuscript. All authors contributed to the manuscript revision, read, and approved the submitted version.
