# Supplementary material for: Differences and similarities in personality functioning across different types of eating disorders
Source: Front Psychiatry. 2023 Jun 1;14:1155725. doi: 10.3389/fpsyt.2023.1155725 (PMC10267354; doi:10.3389/fpsyt.2023.1155725)
Supplement: Supplementary file 1 [file Table_1.pdf]

Supplement

Associations between personality structure (OPD-SQ) and eating disorder symptoms - best predictors in models of the global scale and main scales.

**Model**	**F / t**	stand. β	p	adjusted R^2
OPD-SQ Global Scale	14.859	-	< .001	.389
Munich ED-Quest total score	6.668	.650	< .001	-
Self-perception	12.379	-	< .001	.343
Consumption of high-calorie foods during binge eating (Munich ED-Quest)	5.618	.489	< .001	-
Object-perception	11.973	-	< .001	.337
Feeling pressured by other people's expectations (Munich ED-Quest)	5.729	.499	< .001	-
Self-regulation	9.527	-	< .001	.281
Munich ED-Quest total score	3.628	.449	< .001	-
Regulation of relationships	6.999	-	.000	.216
Feeling out of control of one's life (Munich ED-Quest)	3.721	.360	,000	-
Internal communication	10.275	-	< .001	.311
Shape concern (EDE-Q Scale)	3.500	.390	.001	-
External communication	2.151	-	.065	.051
Increased anxiety (Munich ED-Quest)	3,188	.304	,002	-
Attachment to internal objects	6.999	-	.000	.252
Feeling pressured by other people's expectations (Munich ED-Quest)	3.721	.360	.000	-
Attachment to external objects	11.630	-	< .001	.340
Consumption of high-calorie foods during binge eating (Munich ED-Quest)	2,177	.377	.032	-

F = Analysis of Variance F-statistics, t = t-test t-statistics, p = significance
